# The Effectiveness of a Computer-Tailored E-Learning Program for Practice Nurses to Improve Their Adherence to Smoking Cessation Counseling Guidelines: Randomized Controlled Trial

**DOI:** 10.2196/jmir.9276

**Published:** 2018-05-22

**Authors:** Dennis de Ruijter, Math Candel, Eline Suzanne Smit, Hein de Vries, Ciska Hoving

**Affiliations:** ^1^ Care and Public Health Research Institute Department of Health Promotion Maastricht University Maastricht Netherlands; ^2^ Care and Public Health Research Institute Department of Methodology & Statistics Maastricht University Maastricht Netherlands; ^3^ Amsterdam School of Communication Research Department of Communication Science University of Amsterdam Amsterdam Netherlands

**Keywords:** online learning, guideline adherence, advanced practice nursing, randomized controlled trial, smoking cessation

## Abstract

**Background:**

Improving practice nurses’ (PN) adherence to smoking cessation counseling guidelines will benefit the quality of smoking cessation care and will potentially lead to higher smoking abstinence rates. However, support programs to aid PNs in improving their guideline uptake and adherence do not exist yet.

**Objective:**

The aim of this study was to assess the effects of a novel computer-tailored electronic learning (e-learning) program on PNs’ smoking cessation guideline adherence.

**Methods:**

A Web-based randomized controlled trial (RCT) was conducted in which an intervention group (N=147) with full access to the e-learning program for 6 months was compared with a control group (N=122) without access. Data collection was fully automated at baseline and 6-month follow-up via online questionnaires, assessing PNs’ demographics, work-related factors, potential behavioral predictors based on the I-Change model, and guideline adherence. PNs also completed counseling checklists to retrieve self-reported counseling activities for each consultation with a smoker (N=1175). To assess the program’s effectiveness in improving PNs’ guideline adherence (ie, overall adherence and adherence to individual counseling guideline steps), mixed linear and logistic regression analyses were conducted, thus accommodating for the smokers being nested within PNs. Potential effect moderation by work-related factors and behavioral predictors was also examined.

**Results:**

After 6 months, 121 PNs in the intervention group (82.3%, 121/147) and 103 in the control group (84.4%, 103/122) completed the follow-up questionnaire. Mixed linear regression analysis revealed that counseling experience moderated the program’s effect on PNs’ overall guideline adherence (beta=.589; 95% CI 0.111-1.068; *P*_Holm-Bonferroni_ =.048), indicating a positive program effect on adherence for PNs with a more than average level of counseling experience. Mixed logistic regression analyses regarding adherence to individual guideline steps revealed a trend toward moderating effects of baseline levels of behavioral predictors and counseling experience. More specifically, for PNs with less favorable scores on behavioral predictors (eg, low baseline self-efficacy) and high levels of counseling experience, the program significantly increased adherence.

**Conclusions:**

Results from our RCT showed that among PNs with more than average counseling experience, the e-learning program resulted in significantly better smoking cessation guideline adherence. Experienced PNs might have been better able to translate the content of our e-learning program into practically applicable counseling strategies compared with less experienced colleagues. Less favorable baseline levels of behavioral predictors among PNs possibly contributed to this effect, as there was more room for improvement by consulting the tailored content of the e-learning program. To further substantiate the effectiveness of e-learning programs on guideline adherence by health care professionals (HCPs), it is important to assess how to support a wider range of HCPs.

**Trial Registration:**

Netherlands Trial Register NTR4436; http://www.trialregister.nl/trialreg/admin/rctview.asp?TC=4436 (Archived by WebCite at http://www.webcitation.org/6zJQuSRq0)

## Introduction

Smoking is the most preventable cause of illness and premature death worldwide [1,2]. In the Netherlands, 24.1% of adults still smoked in 2016 [3], illustrating the persistent need for effective smoking cessation strategies. For example, the general practice setting has great potential for cessation support, as over 75% of Dutch smokers visit their general practice at least once a year [4]. General practice health care professionals (HCPs) such as practice nurses (PN) and general practitioners (GPs) are trained to use evidence-based smoking cessation guidelines (ie, the STIMEDIC guideline [5] being the most recent one) to counsel their smoking patients. Applying such guidelines in structured cessation treatment, which combines behavioral and pharmacological support [5], is known to have beneficial effects on smokers’ abstinence rates [6]. However, only in 25% to 33% of consultations do smokers receive a quit smoking advice in their general practice [4]. Moreover, once a quit advice has been given, more extensive smoking cessation support should be provided, which is most often the responsibility of PNs [7]. Yet, also concerning subsequent steps of evidence-based smoking cessation guidelines, PNs’ adherence is suboptimal [8]. Consequently, improving PNs’ guideline adherence would benefit the quality of the smoking cessation care in the general practice and could therefore lead to higher smoking abstinence rates [9,10].

An earlier study investigating PNs’ needs for guideline adherence support found that they were interested in an individually relevant, easy-to-use, and practically applicable program or intervention [11]. Moreover, research showed that PNs’ guideline adherence is positively related to their level of self-efficacy for using a guideline and perceiving advantages of using a guideline [8,12-14]. Such behavioral predictors could be targeted through intervention programs aimed to improve PNs’ guideline adherence. More specifically, providing PNs with content tailored to behavioral predictors fulfills their need for an individually relevant program, and therefore, tailored content is more likely to be read and remembered, compared with nontailored program content [15]. For instance, tailored content can be matched with PNs’ individual level of self-efficacy and their perceived advantages of guideline usage: information can be provided regarding potentially difficult counseling situations (eg, when limited time is available) or regarding specific benefits of using a smoking cessation guideline during consultations (eg, increasing counseling quality), as identified by each individual PN in an earlier evaluation [16].

Additionally, providing PNs with online access to tailored content (ie, computer-tailored, CT) enables them to consult it time-efficiently and whenever and wherever they desire [17,18]. Previously tested CT programs proved to be effective in changing various (determinants of) health behaviors, including smoking cessation [19,20]. Therefore, by targeting PNs’ behavioral predictors via a Web-based CT support program, positive behavior change can be achieved among PNs, meaning that they improve their smoking cessation guideline adherence. Moreover, despite PNs’ interest in tailored adherence support [11], such (Web-based) CT programs do not yet exist with the aim to increase PNs’ smoking cessation guideline adherence. Therefore, we developed and tested a novel Web-based CT electronic learning (e-learning) program for PNs to support them to improve their smoking cessation counseling guideline adherence [21].

The aim of the study described here was to assess the effects of the CT e-learning program on PNs’ smoking cessation guideline adherence in a randomized controlled trial (RCT). We hypothesized that PNs’ guideline adherence would significantly improve as a result of exposure to the CT e-learning program.

## Methods

### Study Design

We conducted an RCT to investigate the effectiveness of the CT e-learning program on PNs’ smoking cessation guideline adherence, compared with no intervention. A full description of the design of the RCT can be found elsewhere [21]. Evaluation by the Medical Ethics Committee Atrium-Orbis-Zuyd (14-N-17) revealed that no medical ethical clearance for this study was needed according to the rules of the Medical Research Involving Human Subjects Act (WMO). The study is registered with the Dutch Trial Register (NTR4436).

### The Computer-Tailored E-Learning Program

The CT e-learning program was structurally based on previously developed CT programs [22,23] and consisted of (1) Several e-learning modules in which PNs had access to individually tailored advice, a forum, and smoking cessation counseling materials (both to inform themselves and to provide to smokers) and (2) Three general modules with project information, frequently asked questions about the RCT, and a counseling checklist to monitor self-reported counseling activities during the trial [21]. The content of advice modules was tailored to several respondent characteristics theoretically grounded in the I-Change Model (ICM [24]), which were previously demonstrated to be effective in achieving behavior change [25-28]: demographics (eg gender), premotivational factors (eg, knowledge), motivational factors (eg, self-efficacy), postmotivational factors (eg, coping planning), intention (to use a smoking cessation guideline), and behavior (ie, self-reported application of smoking cessation guideline steps).

### Participants and Procedure

PNs across the Netherlands were contacted through email, newsletters, and website messages via national organizations for PNs or primary care professionals in general, as well as via a project website [29] and social media platforms (ie, Twitter, LinkedIn, and Facebook). Additionally, individual PNs were contacted by the research team via telephone through their general practice. Eligible PNs were actively engaged in smoking cessation counseling in a Dutch general practice, had Internet access and an active email account, and were sufficiently proficient in Dutch. Upon interest and obtaining important project information via telephone and email, PNs were prompted to visit the CT e-learning program to complete an online informed consent form, were randomized (ie, allocation by a computer software randomization device), and were asked to fill out the Web-based baseline questionnaire. As PN enrollment in the trial was spread over a period of 6 months, randomization was conducted at respondent level at the time of enrollment of an individual PN.

Individual PNs who were randomly allocated to the intervention group of the trial had access to all e-learning and general modules described above and received a tailored feedback letter based on their answers to the baseline questionnaire; this letter provided individual PNs with a summary of various pieces of tailored advice (ie, on different motivational factors and behavior) and instructions on where to find more elaborate advice in the e-learning modules. PNs in the control group only had access to the general modules. During a 6-month time period (ie, upon completion of the baseline questionnaire), PNs in the intervention and control group were free to visit the modules of the CT e-learning program that were available to them based on their group allocation as many times as they wanted. PNs could directly print content from the modules and save this content on their computer.

**Figure 1:**
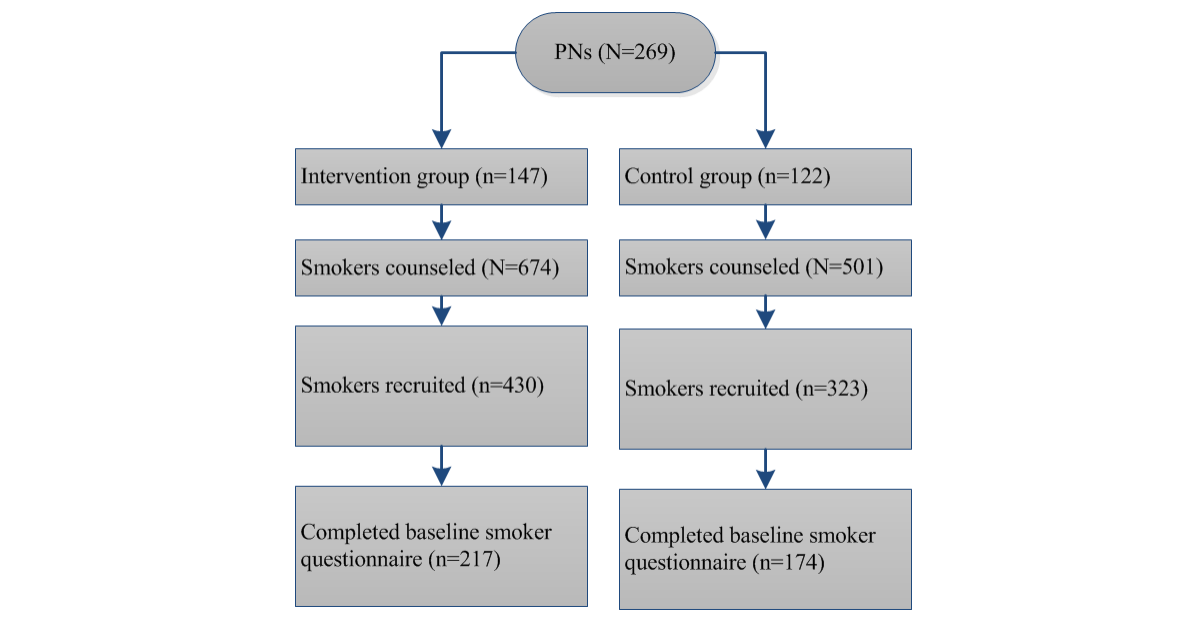
Flow of counseled smokers recruited by practice nurses.

During the trial, PNs in both the intervention and control group were asked to engage in smoking cessation counseling with their smoking patients when the opportunity arose. All PNs were asked to recruit these smokers to partake in the trial (Figure 1). When smokers agreed, PNs were instructed to record smokers’ date of birth and email address. Smokers then directly received an email invitation to participate in the trial and to fill out an online questionnaire.

### Data Collection Among Practice Nurses

Baseline and 6-month follow-up questionnaires for PNs were informed by the ICM [24] and were based on questionnaires previously used among HCPs to assess smoking cessation activities [13,23,30]. Questionnaires were identical for intervention and control group PNs and administered in a Web-based format. The baseline questionnaire for PNs consisted of questions concerning demographic characteristics, potential behavioral predictors of adherence, and their guideline adherence. The follow-up questionnaire included the same questions about potential behavioral predictors and PNs’ guideline adherence. Additional data on PNs’ smoking cessation guideline adherence were collected via the counseling checklist that PNs filled out after each consultation with a smoker throughout the 6-month intervention period and during a 6-month follow-up period.

#### Demographics

After providing online informed consent, every PN was requested to fill out their first and last name, gender, date of birth, and smoking status (smoker, ex-smoker, nonsmoker). Subsequently, they filled out in how many general practices they worked, how many hours they worked per week, and whether or not they were listed in the Dutch Stop Smoking Quality Register (ie, a register with qualified smoking cessation professionals). The final questions concerned the practice in which a PN worked most hours per week; they filled out practice name, experience in smoking cessation counseling in years, the presence of designated smoking cessation consulting hours (yes or no), and whether patients’ smoking status was systematically registered in their patient files (yes or no).

#### Behavioral Predictors

Several socio-cognitive factors were assessed as potential predictors of guideline adherence, informed by the ICM [24]: intention, knowledge, attitude, self-efficacy, social influence, action planning, and coping planning. Items were based on previously used questionnaires about behavioral predictors related to nurses’ smoking cessation counseling [13,19,30].

PNs’ intention to use a smoking cessation guideline was assessed by two questions addressing the intention to use (1) Any evidence-based smoking cessation guideline and (2) The most recent Dutch counseling protocol, specifically (ie, STIMEDIC guideline [5]: *Do you intend to use the STIMEDIC quit smoking guideline?*), using the same answering scale (1=definitely not, 4=do not know, 7=definitely).

PNs’ knowledge of evidence-based smoking cessation guidelines was assessed by 18 true-false items concerning the content of the STIMEDIC guideline (eg, *the first consultation of a smoking cessation trajectory starts with providing a quit advice)*. As such, PNs scored points for every statement they appropriately identified to correctly reflect the content of the counseling protocol (range 0-18).

PNs’ attitude was assessed by seven items about perceived advantages (eg, *using an evidence-based guideline improves the quality of my smoking cessation counseling*) and seven items about perceived disadvantages (eg, *using an evidence-based guideline is time-consuming for me*) of using an evidence-based smoking cessation guideline (1=completely disagree, 5=completely agree). These items were subsequently combined into separate scales for perceived advantages (Cronbach alpha=0.82; Ω=0.82) and perceived disadvantages (Cronbach alpha=0.74; Ω=0.74).

PNs’ level of self-efficacy was assessed by ten items describing potentially difficult situations when trying to adhere to an evidence-based smoking cessation guideline (eg, *when it is very busy at the general practice*) and asking PNs how difficult they would find it to follow a guideline in each of these situations (1=very difficult, 5=very easy). All items were combined into a self-efficacy scale (Cronbach alpha=0.84; Ω=0.84).

PNs’ perceived social influence was assessed by three items about social modeling (eg, *the GP works with an evidence-based smoking cessation guideline*), five items about social support (eg, *colleagues in other practices support the use of an evidence-based smoking cessation guideline*), and five items about social norms (eg, *the practice manager thinks using an evidence-based smoking cessation guideline is important*). All items assessed the potential influence of important others within and outside the general practice (1=completely disagree, 3=neutral, 5=completely agree) and were subsequently combined into separate scales for social modeling (Cronbach alpha=0.62; Ω=0.63), social support (Cronbach alpha=0.73; Ω=0.74), and social norms (Cronbach alpha=0.71; Ω=0.72). Scores that represented a *not applicable* answering category were assigned a neutral score.

PNs’ intention to make action plans and coping plans was assessed by eight and ten items, respectively (yes or no). Action plans addressed specific activities for preparing a smoking cessation consultation with a patient (eg, *discussing a patient’s smoking status with the GP*), whereas coping plans addressed their aspiration to develop a concrete plan for dealing with potentially difficult situations (ie, plans for dealing with the same potentially difficult situations as assessed in the self-efficacy questions). Subsequently, sum scores for both action plans (range 0-8) and coping plans (range 0-10) were computed.

#### Guideline Adherence

Questions on guideline adherence concerned the nine evidence-based counseling steps, as described in the STIMEDIC guideline [5]: (1) advising to quit smoking, (2) assessing smoking profile and smoking history, (3) assessing motivation to quit, (4) increasing motivation, (5) assessing barriers to quitting, (6) discussing barriers, (7) informing about cessation aids, (8) making a quit plan and setting a quit date, and (9) arranging follow-up after the quit date. PNs’ adherence at baseline was assessed by asking PNs to self-report their adherence to each guideline step (eg, *I advised my patient to quit smoking; step 1*) during complete smoking cessation trajectories (ie, intake and follow-up consultations) of their last ten patients (range 0-10). These data on PNs’ adherence were used to create CT advice for PNs in the intervention group regarding their behavior. In the effect analyses, PNs’ baseline guideline adherence score was used as a covariate. Additionally, during the trial period, guideline adherence was assessed by asking PNs to self-report their adherence to each guideline step (ie, *Please select which subjects were addressed during the consultation with your smoking patient*) after every consultation with a smoking patient (yes or no) using the counseling checklist (ie, one of the general modules in the CT e-learning program available for PNs in both the intervention and control group). This resulted in a score from 0 (none of the steps were adhered to) to 9 (all steps were adhered to) for each individual consultation with a smoker. Checklists of consultations with the same smoker were combined into a single score for guideline adherence, reflecting a PN’s adherence during a complete counseling trajectory of a smoking patient, which was the primary outcome measure in the effect analyses.

### Sample Size Calculation

We calculated the required sample size based on the possibility to detect a difference of medium effect size (ie, adherence to two additional guideline steps) between intervention and control group PNs ( alpha=5%; beta=10%). As a result, at least 95 PNs per condition at the end of the trial would be sufficient [21]. However, to detect a medium effect size for an interaction with the intervention factor when assuming an intraclass correlation of .25, at least 105 PNs per condition are needed to ensure a statistical power of 80%. Considering 30% attrition, we aimed to include 300 PNs at baseline.

### Statistical Analyses

Reliability analyses (ie, Cronbach alpha and Ω) were conducted using R version 3.4.0 (R Foundation for Statistical Computing), and other statistical analyses were conducted using SPSS version 23.0 (IBM Corp). Descriptive analyses were conducted to summarize PNs’ characteristics, whereas independent-samples *t* tests and chi-square tests determined significant (*P*<.05) baseline differences between intervention and control group PNs. Logistic regression was used to determine selective dropout of PNs after baseline, including variables potentially related to PNs’ guideline adherence (ie, specific work-related variables, behavioral predictors, and baseline guideline adherence). On the basis of analyses for baseline differences and selective dropout, statistically significant variables were identified and included as covariates in further analyses.

As smokers were nested within PNs participating in the trial, mixed regression analyses were conducted to assess the effects of exposure to the CT e-learning program on PNs’ smoking cessation guideline adherence. Both PNs’ overall adherence score (range 0-9) was used as outcome measure and their adherence score for each guideline step separately (ie, step-based adherence; 0=nonadherent, 1=adherent). Therefore, both linear and logistic mixed models were run, including the same covariates. Effect moderators were tested by including interaction effects with PNs’ group allocation (ie, intervention or control) to the regression models tested. On the basis of literature, several work-related factors (ie, counseling experience and presence of consulting hours [31,32]) and behavioral predictors (ie, intention, attitude, self-efficacy, and social influence [8,33,34]), potentially moderating the program’s effect on PNs’ adherence, were tested. First, nonsignificant interaction effects were stepwise deleted using a backward deletion procedure, meaning that at each step the least significant interaction effect was removed. Second, nonsignificant covariates were deleted from the model following the same procedure, with the restriction that these covariates remained in the model if they were also part of a significant interaction term. Upon finding a significant interaction effect, subsequent subgroup analyses were conducted to determine the nature of the moderation using adjusted alpha levels (Holm-Bonferroni method) to correct for multiple testing. For subgroup analyses, the final mixed regression model was repeated, while replacing the original moderator with three centered versions of the moderator, centered by subtracting the mean − 1 SD, the mean, and the mean + 1 SD from the original scores on the moderating variable. This allows for testing the effects of the e-learning program for three subgroups: one group corresponding to a score of the mean − 1 SD on the moderating variable, a second group with a score at the average on the moderating variable, and a third group with a score at mean + 1 SD on the moderating variable.

As 211 PNs (78.4%, 211/269) completed at least one checklist (which was needed to calculate the primary outcome measure), it meant that 58 PNs were excluded from effect analyses. For this reason, sensitivity analyses were conducted by replacing missing values on the primary outcome measure (guideline adherence) with scores assuming some dependency between the score being missing and the adherence score itself, either following an optimistic or a pessimistic scenario. In both scenarios, missing data were imputed for these 58 PNs based on the average number of patients counseled during the trial per PN. Furthermore, PNs’ dropout status was taken into account, as some PNs, who did not complete any checklists, also did not fill out the follow-up questionnaire (ie, PN dropouts). One might expect PNs who did complete the follow-up questionnaire (ie, retained PNs) to be more motivated and to be more adherent if they would have completed the checklists during the intervention period. This was taken into account when imputing data. In the optimistic imputation scenario, retained PNs were assumed to be adherent in 90% of the consultations with smokers, whereas PN dropouts were assumed to be adherent in only 80% of their consultations. In the pessimistic scenario, a 50% probability of adherence was assumed for retained PNs and a 20% probability of adherence for PN dropouts. The datasets obtained under these two imputation scenarios were analyzed with the mixed regression models, as obtained after backward deletion of nonsignificant (interaction) effects in the analysis of only the complete cases.

## Results

### Sample Characteristics

**Figure 2:**
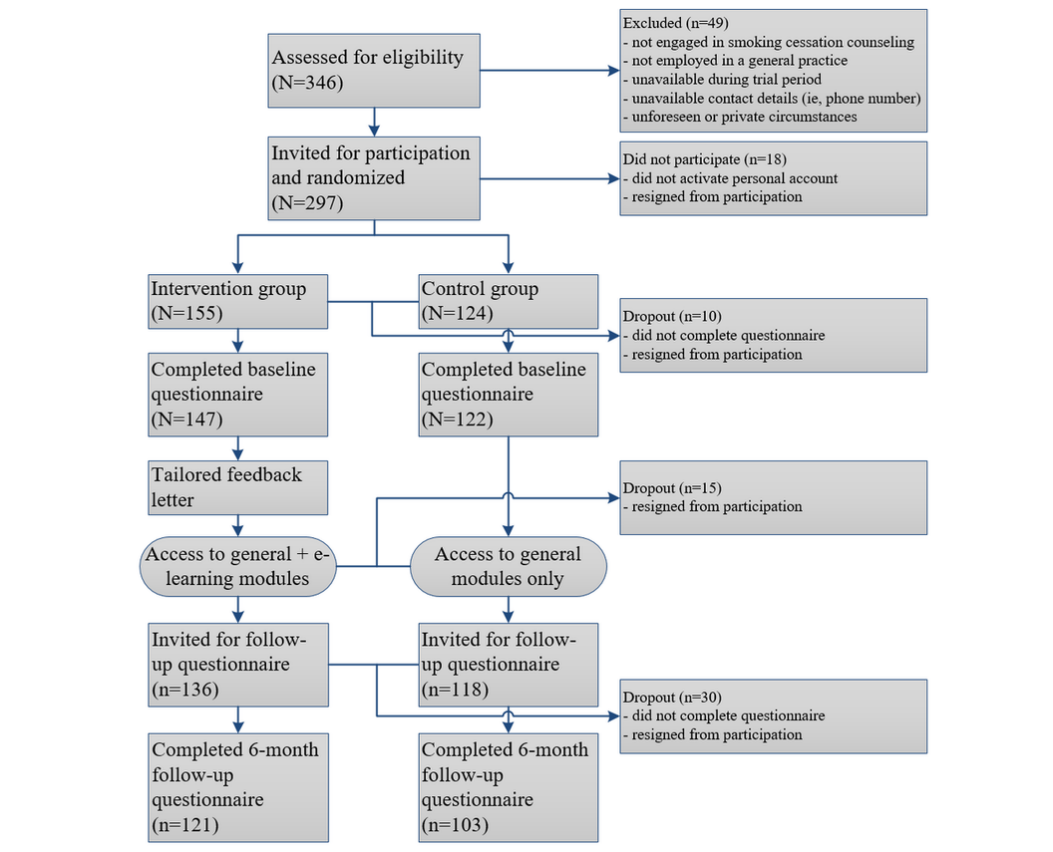
Flow and randomization of practice nurses that were recruited from January 2016 to June 2016.

Figure 2 shows the flow of PNs included in the trial from initial assessment of eligibility to randomization and completion of baseline and follow-up questionnaires. Of the 346 PNs assessed for eligibility, 49 (14.2%) did not meet inclusion criteria, and 18 (5.2%) refrained from participation. After randomization, 147 (49.5%, 147/297) and 122 (41.1%, 122/297) PNs were allocated to the intervention and control group, respectively, and completed the baseline questionnaire. Unequal group sizes are the result of chance, as randomization took place at respondent level, and each PN had a 50% probability of being allocated to either group.

The baseline sample of PNs (Table 1) had a mean age of 47.3 years (range 23-66), the vast majority was female (97.8%, 263/269), and very few PNs (1.1%, 3/269) were current smokers. Nearly half (47.2%, 127/269) worked in more than one general practice, and PNs worked on average almost 26 hours a week (range 3-42). Many PNs (66.9%, 180/269) were listed in the Dutch Stop Smoking Quality Register, and the mean-reported PN counseling experience was 5.6 years (range 0-20). Finally, almost half of the PNs (47.6%, 128/269) worked in a general practice with designated smoking cessation consulting hours, and nearly all (92.6%, 249/269) reported to systematically register their patients’ smoking status in their patient files. During the trial, PNs engaged in smoking cessation counseling with 5.6 different patients on average (range 1-26).

Baseline characteristics of PNs were comparable between intervention and control group, except for the presence of designated smoking cessation consulting hours, which was significantly more often reported by PNs in the control group (χ^2^_1_=10.1; *P*=.001). Therefore, the presence of designated consulting-hours was included as a covariate in all effect analyses.

### Attrition Analyses

After 6 months, 254 PNs remained in the trial and were invited for the follow-up measurement, which was completed by 88.9% (121/136) of intervention group and 87.3% (103/118) of control group PNs, respectively (Figure 2). Attrition analyses revealed that PNs who completed the follow-up measurement had a higher baseline intention to use the STIMEDIC guideline (odds ratio, OR 1.41, 95% CI 1.00-1.98) and had more baseline knowledge about the STIMEDIC guideline content (OR 1.39, 95% CI 1.07-1.82) compared with PNs who dropped out before the follow-up measurement (Table 2). Therefore, these two variables were also included as covariates in all effect analyses.

### Effect Analyses

#### Overall Adherence

Table 3 shows the mixed regression results on PNs’ overall guideline adherence; mean-centered values are reported for variables included in interaction effects to enable meaningful interpretation of the effect of *group* (eg, in Table 3, the effect of group allocation illustrates the effect of the intervention for PNs that score average on counseling experience). The results reveal a significant interaction effect of group allocation with counseling experience (*P*=.045) and a main effect of perceived advantages of guideline use (*P*=.03). Subgroup analyses showed that for PNs with more than average counseling experience (ie, mean + 1SD = 9.4 years of experience), allocation to the intervention group (ie, access to the CT e-learning program) resulted in a significantly higher overall adherence compared with the control group (beta=.589; 95% CI 0.111-1.068; *P*_Holm-Bonferroni_=.048). The subgroup analysis for less experienced PNs (ie, mean 5.6 years of experience and mean − 1SD = 1.9 years of experience) revealed no significant intervention effect.

**Table 1:** Characteristics of practice nurses and comparison of characteristics between intervention and control groups.

Characteristics		Overall sample (N=269)	Intervention group (n=147)	Control group (n=122)	Chi-square	T (degrees of freedom)	*P* value
Age in years, mean (SD)		47.3 (9.5)	48.0 (9.6)	46.5 (9.4)	N/A^a^	−1.345 (267)	.18
Female, n (%)		263 (97.8)	143 (97.3)	120 (98.4)	X^2^_1_=0.4	N/A	.55
**Smoking status, n (%)**					X^2^_2_=0.4	N/A	.84
	Nonsmoker	151 (56.1)	84 (57.1)	67 (54.9)			
	Ex-smoker	115 (42.8)	61 (41.5)	54 (44.3)			
	Smoker	3 (1.1)	2 (1.4)	1 (0.8)			
Employed in >1 practice, n (%)		127 (47.2)	70 (47.6)	57 (46.7)	X^2^_1_=0.0	N/A	.88
Working hours, mean (SD)		25.7 (7.4)	25.7 (7.5)	25.7 (7.4)	N/A	−0.007 (267)	.99
Registration in Stop Smoking Quality Register, n (%)		180 (66.9)	95 (64.6)	85 (69.7)	X^2^_1_=0.8	N/A	.38
Counseling experience in years, mean (SD)		5.6 (3.7)	5.5 (3.6)	5.8 (3.8)	N/A	-0.602 (267)	.55
Consulting hours present, n (%)		128 (47.6)	57 (38.8)	71 (58.2)	X^2^_1_=10.1	N/A	.001
Registration of smoking status in patient files, n (%)		249 (92.6)	135 (91.8)	114 (93.4)	X^2^_1_=0.3	N/A	.62
Number of counseled patients during the trial, mean (SD)		5.6 (4.4)	5.8 (4.1)	5.3 (4.8)	N/A	-0.700 (209)	.49

^a^N/A: not applicable.

**Table 2:** Baseline characteristics of practice nurses (PN) and their odds to predict PN retention.

Characteristics	Mean (SD)	Range	Odds ratio (95% CI)	*P* value
Working hours	25.7 (7.4)	3-42	0.95 (0.91-1.00)	.06
Counseling experience in years	5.6 (3.7)	0-20	0.96 (0.88-1.06)	.42
Intention to use any evidence-based guideline (1-7)	6.3 (0.8)	4-7	0.86 (0.51-1.45)	.57
Intention to use STIMEDIC (1-7)	5.5 (1.1)	2-7	1.41 (1.00-1.98)	.049
STIMEDIC knowledge (0-18)	14.6 (1.3)	11-18	1.39 (1.07-1.82)	.02
Perceived advantages (1-5)	4.2 (0.6)	2-5	0.80 (0.41-1.56)	.52
Perceived disadvantages (1-5)	1.9 (0.6)	1-4	0.56 (0.28-1.13)	.10
Self-efficacy (1-5)	2.8 (0.6)	1.3-5	0.62 (0.34-1.13)	.12
Social modeling (1-5)	3.2 (0.9)	1-5	0.70 (0.38-1.29)	.25
Social support (1-5)	3.3 (0.8)	1-5	1.61 (0.60-4.28)	.35
Social norms (1-5)	1.4 (0.7)	1.4-5	1.34 (0.47-3.83)	.58
Action planning (0-8)	6.4 (1.7)	0-8	0.97 (0.78-1.22)	.82
Coping planning (0-10)	6.7 (2.7)	0-10	1.01 (0.89-1.16)	.86
Baseline guideline adherence (0-9)	8.5 (1.6)	0-10	1.06 (0.86-1.31)	.59

**Table 3:** Results of backward linear mixed regression analysis on practical nurses’ overall guideline adherence.

Final model	Coefficient	95% CI	*P* value
Group (control=0; intervention=1)	.245	−0.087 to 0.577	.19
Counseling experience	−.046	−0.113 to 0.021	.18
Perceived advantages	.319	0.031-0.608	.03
Group^*^counseling experience	.092	0.002-0.183	.045

#### Step-Based Adherence

Multimedia Appendix 1 shows the results on PNs’ step-based adherence for each step separately; again mean-centered values are reported to enable meaningful interpretation of the effect of *group* in the presence of a significant interaction. Regarding adherence to *step 1* (ie, advising to quit smoking), a significant main effect was found of PNs’ baseline adherence to step 1 (*P*=.002). Regarding adherence to *step 2* (ie, assessing smoking profile and smoking history), a significant interaction effect of group allocation with perceived advantages of guideline use (*P*=.001) was identified. Regarding adherence to *step 3* (ie, assessing motivation to quit), an interaction effect with self-efficacy (*P*=.02) was found. Regarding adherence to *step 4* (ie, increasing motivation), a borderline significant interaction effect of group allocation with counseling experience (*P*=.06) was found, as well as main effects of baseline adherence to step 4 (*P*=.01) and of perceived advantages of guideline use (*P*=.03). Regarding adherence to *step 5* (ie, assessing barriers to quitting), significant interaction effects of group allocation with social modeling (*P*=.009) and with social support were found (*P*=.046). Regarding adherence to *step 6* (ie, discussing barriers), a significant main effect was found of perceived advantages of guideline use (*P*=.045). Regarding adherence to *step 7* (ie, informing about cessation aids), significant interaction effects of group allocation with perceived disadvantages (*P*=.01) and with self-efficacy (*P*=.001) and a borderline significant interaction effect with counseling experience (*P*=.05) were found. Furthermore, a significant main effect of social modeling (*P*=.01) was found. Regarding adherence to *step 8* (ie, making a quit plan and setting a quit date), significant interaction effects of group allocation with counseling experience (*P*=.01), with social support (*P*=.001), and with social norms (*P*=.005) were found. Concerning adherence to *step 9* (ie, arranging follow-up after the quit date), an interaction with social support (*P*=.04) was found.

Detailed results on subgroup analyses for each significant interaction effect are presented in Multimedia Appendix 2. Overall, the results show that higher adherence scores on individual guideline steps of intervention group PNs, compared with control group PNs, occur for (1) High levels of counseling experience and perceived social support and (2) Low levels of perceived advantages, perceived disadvantages, self-efficacy and social modeling, and little social norms.

#### Sensitivity Analyses

After replicating the final mixed regression models following an *optimistic imputation scenario*, results for overall guideline adherence indicated that only the main effect of perceived advantages remained significant. Regarding step-based adherence, similar results were found for adherence to guideline steps 1, 2, and 8. Concerning adherence to steps 5 and 7 only, the interaction effects with social modeling and self-efficacy could be replicated, and concerning adherence to step 4, only the main effect of perceived advantages remained significant. Main and interaction effects concerning guideline steps 3, 6, and 9 were no longer or only marginally significant after conducting these optimistic sensitivity analyses.

After replicating the final mixed regression models following a *pessimistic imputation scenario*, main and interaction effects for overall adherence were no longer or only marginally significant. Regarding step-based adherence, similar results were found for adherence to guideline steps 1, 6, 8, and 9, whereas regarding step 4, only main effects of baseline adherence to step 4 and perceived advantages remained significant. Main and interaction effects concerning guideline steps 2, 3, 5, and 7 were no longer or only marginally significant after conducting these pessimistic sensitivity analyses.

## Discussion

### Principal Findings

The present RCT tested the effectiveness of a novel CT e-learning program for PNs to improve their adherence to evidence-based smoking cessation guidelines in Dutch general practice. Our results suggest that among PNs with more than average smoking cessation counseling experience, access to the CT e-learning program resulted in significantly better guideline adherence. A more detailed inspection of PNs’ guideline adherence revealed comparable results for several specific guideline steps (ie, increasing motivation, discussing cessation aids, and making a quit plan). Additionally, a trend was observed that for PNs reporting less favorable baseline levels for behavioral predictors such as self-efficacy and favorable levels of perceived social support, the e-learning program was effective in improving guideline adherence.

Regarding PNs’ counseling experience, subgroup analyses illustrated that more experienced PNs in the intervention group adhered significantly better to the guideline (ie, difference of adherence to 0.6 steps) at follow-up compared with equally experienced PNs in the control group. This finding means that the CT e-learning program successfully promoted guideline adherence among PNs with more counseling experience, which is in line with the fact that a significant positive association between counseling experience and PNs’ baseline guideline adherence was found (Multimedia Appendix 3). Experienced PNs might have been better able to practically apply the content of the e-learning program during their counseling, as they have likely dealt with many different types of smokers and difficult counseling situations in the past. Due to these past coping experiences, it could have been easier for them, compared with less experienced PNs, to translate the theoretically grounded e-learning content to counseling situations encountered in practice, leading to improved guideline adherence. Yet, evidence about the influence of HCPs’ counseling experience on their application of evidence-based guidelines is scarce. One study among physicians investigated the association between work experience and guideline adherence after taking an e-learning course but could not establish such an association [35]. Another study focused on the relation between work experience and knowledge and found that more experienced professionals scored higher on knowledge after using a nontailored interactive video intervention [36]. However, an association of experience with knowledge was not established among PNs in the present trial (Multimedia Appendix 3), and neither was it found in a study about the effects of an e-learning course on the knowledge level of nursing staff [37]. Hence, an alternative explanation could be considered; perhaps experienced PNs were able to spend their counseling time more efficiently, giving them opportunity to visit the CT e-learning program more often (eg, in between consultations) and—as a consequence—benefit more from its content. However, although more frequent users were more adherent in the present trial, program usage (mean number of module visits 3.2, SD 6.2, range 0-48) was not significantly associated with PNs’ guideline adherence, nor did we identify that more experienced PNs were more frequent users (data not shown). This is comparable with a study among GPs, in which factors such as working experience were not predictive of usage of an e-learning program to promote dementia guideline adherence [38]. It is important to further investigate if and why HCPs, including PNs, with more counseling experience benefit more from CT e-learning programs. For example, process evaluation of users’ interaction with a program can aid our understanding of the working mechanisms (eg, targeted behavioral predictors) of exposure to tailored program content [39]. Such insights could subsequently be used to improve a program’s effectiveness for individuals with varying levels of counseling experience (eg, by additionally tailoring program content on an individual’s level of experience).

Results regarding PNs’ step-based guideline adherence illustrated that, besides counseling experience, several behavioral predictors could explain better adherence scores of PNs in the intervention group compared with the control group. PNs’ perceived advantages and disadvantages of guideline application, level of self-efficacy, social modeling, social norms, and social support at baseline all moderated the e-learning program’s effect on PNs’ step-based adherence. Although moderation effects could not be replicated precisely for each separate guideline step, a trend was observed that especially a high level of baseline social support and lower baseline levels of the other predictors were related to better effects of the program on adherence. As the content of the e-learning program was designed to especially target such behavioral predictors of guideline adherence [21], it is likely that intervention group PNs, initially scoring less favorable on these predictors, were more able to significantly improve their adherence through progress made on these behavioral predictors. An analysis of PNs’ change scores concerning these behavioral predictors, indeed found that intervention group PNs had more favorable scores compared with control group PNs, indicating more progress in terms of their levels of perceived advantages, disadvantages, self-efficacy, social modeling, social norms, and social support (data not shown). Although these change scores were not statistically significant, it is likely that the small improvements on all these predictors together contributed to better step-based adherence scores among intervention group PNs [24].

### Strengths and Limitations

A strength of the present trial is that we enrolled a substantial number of PNs and managed to retain 83.3% (224/269) of them at 6-month follow-up. In comparison, in other studies among nurses, retention rates were considerably lower, eg, studies among hospital nurses that reported 3-month and 6-month follow-up retention rates of 68% and 58% [40], or 56% and 48%, respectively [41]. A high retention rate is essential to obtain adequate power for conducting statistical analyses. Moreover, the PNs in the present trial were able to counsel many smokers during the intervention period, resulting in a large amount of data collected on individual smoking cessation trajectories (N=1175). As each individual PN used the counseling checklist to self-report their application of guideline steps during each consultation with these smokers, we obtained data on PNs’ guideline adherence from at least one consultation per smoker and from on average six different smokers’ counseling trajectories per PN. Our primary outcome measure therefore reflects PNs’ guideline adherence during the entire 12-month trial period instead of at a single time point only (eg, at the end of the intervention period). Outcome measures composed of multiple measurement points are expected to provide a more reliable insight in the target behavior compared with a single measurement point [42]. A final strength is the fact that PNs’ guideline adherence was assessed and analyzed from a step-based perspective, yielding more detailed insights than only taking overall guideline adherence into account.

Nevertheless, we also experienced some challenges during the trial. Our intention was to triangulate data on PNs’ guideline adherence from both smokers’ and PNs’ perspectives to compute the primary outcome measure [21]. Unfortunately, only 33.3% of smokers counseled by PNs (391/1175) also participated in the trial themselves, ie, completed the baseline smoker questionnaire. Qualitative posttrial interviews with PNs (N=17) revealed issues such as time constraints of both smokers and PNs (ie, trial participation was not addressed during the consultation) and smokers’ privacy concerns as reasons for the low participation rate among smokers (data not shown). As a result, conducting effect analyses with these data would be unreliable because of substantial loss of power and selective inclusion of smokers. Moreover, a comparison of smoker-reported data from baseline questionnaires with PN-reported data from counseling checklists revealed significantly higher adherence scores reported by smokers (*t*_390_=−6.73, *P*<.001). As we observed possible ceiling effects in these adherence scores, smoker-reported data were deemed to be unreliable for being used as outcome measure. An explanation could be that smokers overestimated their PNs’ guideline adherence, as they were recruited for participation in the trial by their PN, who also supported them with smoking cessation. Perhaps they were afraid that providing critical answers to questions about their PNs’ performance could influence the relationship with their PN. Although smokers were informed that data were treated anonymously and were not reported back to PNs, the phenomenon of social desirability is often observed when collecting data from patients in a health care setting [43]. It, hence, seems that collecting patient data might not always be reliable and that more objective data collection methods are required. A second limitation was that 58 PNs (21.6%, 58/269) did not manage to recruit and counsel smokers during the intervention period, resulting in lacking data on these PNs’ guideline adherence during consultations. As a result, these PNs could not be included in the effect analyses. Nevertheless, inclusion of data from the remaining PNs (N=211) still ensured adequate power (alpha=5%; beta=10%) to conduct mixed regression analyses concerning their guideline adherence [21]. To examine the sensitivity of these results, the final regression models were repeated following an optimistic and pessimistic imputation scenario. This sensitivity analysis showed that results could have been sensitive to informative dropout (ie, missingness related to unobserved variables), as some main and interaction effects disappeared in both scenarios [44]. Yet, most results were similar to the results of the complete-case analyses, indicating their robustness.

### Implications

In light of the issues described, it would be worthwhile to investigate additional methods to gather data on PNs’ guideline adherence. One such method could be qualitative data collection, by conducting content analyses of audio or video recordings of smoker-PN consultations [45-47]. In the present trial, however, PNs were very reluctant in agreeing to make an audio recording of a smoking cessation consultation, resulting in one or more successful recordings for only 11.5% of PNs (31/269) and 42 recordings in total. During posttrial interviews, PNs reported barriers such as privacy concerns (of both PN and smoker) and the perception that making a recording would influence the interaction with the smoker during a consultation. As a consequence, PNs believed that the recordings would not provide reliable insight into their counseling approach; a phenomenon also described in other recent studies [48,49]. Another potential method to collect data on PNs’ guideline adherence could be to ask PNs to respond to simulated practice situations or clinical vignettes (ie, case studies of smokers visiting their practice for cessation support) to assess their application of evidence-based guideline steps. Earlier studies applied this method, for instance, in written format concerning physiotherapists’ guideline adherence [50] or video-based format concerning suicide guideline implementation [51]. Similarly, PNs could be provided with clinical vignettes that describe different types of smokers in terms of (1) Motivation status (eg, unmotivated, contemplating, and motivated to quit); (2) Perceived barriers toward quitting (eg, smoking partner, weight gain, and stress); and (3) Requests for pharmacotherapy (eg, desire for particular medicine, refusing any pharmacotherapy, and favoring alternative medicine), to assess their guideline adherence in various situations that they could encounter in practice. Research with such clinical vignettes among PNs is needed to determine the potential value of using vignettes to reliably measure PNs’ smoking cessation guideline adherence.

Furthermore, the results of the present trial illustrated that the CT e-learning program successfully improved smoking cessation guideline adherence of experienced PNs. Unfortunately, it was also established that usage of the program by PNs in the trial was quite limited (ie, three module visits on average). This means that the program’s effectiveness could potentially be increased when implementation of the program by PNs (ie, number of modules visits) improves. During posttrial interviews, PNs mentioned that receiving a reminder to visit the program more often could be a potential strategy to stimulate program usage. Furthermore, a process evaluation could inform improving alterations to the program’s tailored content to extend the program’s effectiveness beyond experienced PNs. When such strategies would be combined with program implementation among a larger population of PNs, this could further substantiate the impact of the CT e-learning program on the quality of smoking cessation care in the Netherlands.

### Conclusions

Providing PNs access to a novel CT e-learning program resulted in significantly better adherence to evidence-based smoking cessation guidelines among Dutch PNs experienced in smoking cessation counseling, compared with similar PNs without access to this program. More favorable improvements on behavioral predictors of guideline adherence among intervention group PNs may explain better adherence scores. To further substantiate the effectiveness of e-learning programs on guideline adherence by HCPs, alternative methods of collecting data on guideline adherence should be explored, and strategies are needed to promote program usage and to also support less experienced PNs to adhere to evidence-based smoking cessation guidelines. This could subsequently inform widespread implementation of the e-learning program among PNs.

## Appendix

Multimedia Appendix 1 Results of backward logistic mixed regression analyses on practice nurses’ (PNs’) step-based guideline adherence.

Multimedia Appendix 2 Odds ratios (95% CI) reflecting intervention effects of subgroup analyses of significant interaction effects per guideline step with Holm-Bonferroni corrected P values.

Multimedia Appendix 3 Correlation matrix of practice nurse (PN) characteristics.

Multimedia Appendix 4 CONSORT‐EHEALTH checklist (V 1.6.1).

## Abbreviations

CT: computer-tailored
e-learning: electronic learning
GP: general practitioner
HCP: health care professional
ICM: I-Change Model
OR: odds ratio
PN: practice nurse
RCT: randomized controlled trial
