# Supplementary material for: The Effectiveness of a Computer-Tailored E-Learning Program for Practice Nurses to Improve Their Adherence to Smoking Cessation Counseling Guidelines: Randomized Controlled Trial
Source: J Med Internet Res. 2018 May 22;20(5):e193. doi: 10.2196/jmir.9276 (PMC5989061; doi:10.2196/jmir.9276)
Supplement: Multimedia Appendix 1 [file jmir_v20i5e193_app1.pdf]

Multimedia Appendix 1. Results of backward logistic mixed regression analyses on practice nurses' (PNs') step-based guideline adherence.

Final model		OR (95% CI)	*P* value
**Step 1**			
	Group (control=0; intervention=1)	1.164 (0.705-1.920)	.55
	Baseline adherence step 1	1.199 (1.068-1.345)	.002
**Step 2**			
	Group (control=0; intervention=1)	1.004 (0.652-1.545)	.99
	Perceived advantages	2.658 (1.593-4.437)	.000
	Group[a]* perceived advantages	0.298 (0.143-0.621)	.001
**Step 3**			
	Group (control =0; intervention=1)	2.042 (1.065-3.918)	.03
	Self-efficacy	1.597 (0.813-3.139)	.17
	Group * self-efficacy	0.288 (0.097-0.851)	.02
**Step 4**			
	Group (control=0; intervention=1)	1.347 (0.846-2.144)	.21
	Counseling experience	0.946 (0.863-1.037)	.24
	Baseline adherence step 4	1.148 (1.032-1.277)	.01
	Perceived advantages	1.548 (1.038-2.308)	.03
	Group * counseling experience	1.135 (0.995-1.293)	.06
**Step 5**			
	Group (control=0; intervention=1)	1.294 (0.717-2.336)	.39
	Intention to use STIMEDIC	1.289 (0.985-1.687)	.06
	Social modeling	2.058 (0.995-4.255)	.05
	Social support	0.571 (0.247-1.320)	.19
	Group * social modeling	0.259 (0.095-0.710)	.009
	Group * social support	3.094 (1.019-9.387)	.046
**Step 6**			
	Group (control=0; intervention=1)	1.175 (0.710-1.947)	.53
	Perceived advantages	1.550 (1.010-2.377)	.045
**Step 7**			
	Group (control=0; intervention=1)	0.854 (0.435-1.675)	.65
	Counseling experience	0.891 (0.891-1.001)	.05
	Perceived disadvantages	2.162 (0.792-5.900)	.13
	Self-efficacy	4.353 (1.628-11.638)	.003
	Social modeling	1.654 (1.118-2.446)	.01
	Group * counseling	1.184 (0.997-1.406)	.05
