# Supplementary material for: The Effectiveness of a Computer-Tailored E-Learning Program for Practice Nurses to Improve Their Adherence to Smoking Cessation Counseling Guidelines: Randomized Controlled Trial
Source: J Med Internet Res. 2018 May 22;20(5):e193. doi: 10.2196/jmir.9276 (PMC5989061; doi:10.2196/jmir.9276)
Supplement: Multimedia Appendix 2 [file jmir_v20i5e193_app2.pdf]

Multimedia Appendix 2. Odds ratios (95% CI) reflecting intervention effects of subgroup analyses of significant interaction effects per guideline step with Holm-Bonferroni corrected p-values

Guideline step	Effect of Group (0 = control group, 1 = intervention group) for each of the subgroups					
		P		*P*		*P*
Step 2 smoking profile & history	Low perceived advantages		Medium perceived advantages		High perceived advantages	
	2.084 (1.141–3.809)	.05	1.004 (0.652–1.545)	.99	0.484 (0.257–0.911)	.049*
Step 3 assessing motivation	Low self- efficacy		Medium self-efficacy		High self-efficacy	
	4.441 (1.689-11.678)	.01*	2.042 (1.065-3.918)	.06	0.939 (0.378-2.332)	.89
Step 4 increasing motivation	Low experience		Medium experience		High experience	
	0.841 (0.437-1.620)	.61	1.347 (0.846-2.144)	.42	2.157 (1.080-4.306)	.09
Step 5 assessing barriers	Low social support		Medium social support		High social support	
low social modelling	1.767 (0.753-4.145)	.38	4.220 (1.407-12.657)	.045*	10.085 (1.708-59.442)	.04*
medium social modelling	0.542 (0.200-1.467)	.34	1.294 (0.717-2.336)	.39	3.089 (1.047-9.114)	.12
high social modelling	0.166 (0.031-0.889)	.13	0.397 (0.143-1.104)	.19	0.947 (0.399-2.247)	.90
Step 7 cessation aids	Low self-efficacy		Medium self-efficacy		High self-efficacy	
low counseling experience						
+ low perceived disadvantages	4.402 (1.098-17.643)	.19	1.106 (0.372-3.291)	.86	0.278 (0.073-1.053)	.28
+ medium perceived disadvantages	1.811 (0.586-5.601)	.81	0.455 (0.175-1.187)	.43	0.114 (0.029-0.452)	.02*
+ high perceived disadvantages	0.745 (0.210-2.644)	.65	0.187 (0.052-0.672)	.07	0.047 (0.008-0.265)	.005*
medium counseling experience						
+ low perceived disadvantages	8.257 (2.297-29.677)	.01*	2.075 (0.814-5.292)	.46	0.522 (0.157-1.731)	.86
+ medium perceived disadvantages	3.398 (1.366-8.451)	.06	0.854 (0.435-1.675)	.86	0.215 (0.066-0.701)	.07
+ high perceived disadvantages	1.398 (0.513-3.811)	.86	0.351 (0.128-0.961)	.21	0.088 (0.019-0.408)	.02*
high counseling experience						
+ low perceived disadvantages	15.493 (3.558-67.472)	.003*	3.894 (1.201-12.624)	.14	0.979 (0.244-3.920)	.98
+ medium perceived disadvantages	6.375 (2.127-19.108)	.01*	1.602 (0.652-3.935)	.71	0.403 (0.108-1.502)	.58
+ high perceived disadvantages	2.623 (0.867-7.940)	.38	0.659 (0.219-1.983)	.92	0.166 (0.034-0.811)	.15

Note: for steps 5, 7 and 8 subgroup analyses are conducted concerning multiple moderators. Consequently, two (i.e. step 5) or three moderators (i.e. steps 7 and 8) are used to define 9 resp. 27 subgroups. Low subgroup: score at Mean – 1 SD; Medium subgroup: score at Mean; High subgroup: score at Mean + 1 SD

Multimedia Appendix 2 (continued). Odds ratios (95% CI) reflecting intervention effects of subgroup analyses of significant interaction effects per guideline step with Holm-Bonferroni corrected p-values

Guideline step	Effect of Group (0 = control group, 1 = intervention group) for each of the subgroups					
		P		*P*		*P*
Step 8 quit plan & quit date	Low social norms		Medium social norms		High social norms	
low counseling experience						
+ low social support	0.166 (0.034-0.811)	.10	0.137 (0.042-0.447)	.007*	0.027 (0.003-0.221)	.006*
+ medium social support	4.266 (0.977-18.619)	.11	0.854 (0.411-1.773)	.67	0.171 (0.051-0.568)	.02*
+ high social support	26.532 (2.517-279.675)	.03*	5.309 (1.354-20.826)	.07	1.062 (0.451-2.502)	.89
medium counseling experience						
+ low social support	1.418 (0.588-3.418)	.60	0.284 (0.109-0.742)	.047*	0.057 (0.008-0.380)	.02*
+ medium social support	8.821 (1.987-39.151)	.02*	1.765 (0.996-3.129)	.12	0.353 (0.131-0.950)	.12
+ high social support	54.860 (5.872-617.728)	.007*	10.978 (2.740-43.987)	.006*	2.197 (1.057-4.567)	.15
high counseling experience						
+ low social support	2.934 (0.996-8.643)	.14	0.587 (0.210-1.644)	.52	0.117 (0.018-0.762)	.10
+ medium social support	18.245 (3.342-99.594)	.006*	3.651 (1.553-8.582)	.02*	0.731 (0.253-2.110)	.56
+ high social support	113.476 (8.384-1,535.862)	.003*	22.707 (4.535-113.692)	.001*	4.544 (1.717-12.024)	.01*
Step 9 follow-up	Low social support		Medium social support		High social support	
	0.708 (0.383-1.309)	.54	1.121 (0.735-1.711)	.59	1.777 (0.976-3.234)	.18

Note: for steps 5, 7 and 8 subgroup analyses are conducted concerning multiple moderators. Consequently, two (i.e. step 5) or three moderators (i.e. steps 7 and 8) are used to define 9 resp. 27 subgroups. Low subgroup: score at Mean – 1 SD; Medium subgroup: score at Mean; High subgroup: score at Mean + 1 SD
