# Supplementary material for: The Effectiveness of a Computer-Tailored E-Learning Program for Practice Nurses to Improve Their Adherence to Smoking Cessation Counseling Guidelines: Randomized Controlled Trial
Source: J Med Internet Res. 2018 May 22;20(5):e193. doi: 10.2196/jmir.9276 (PMC5989061; doi:10.2196/jmir.9276)
Supplement: Multimedia Appendix 3 [file jmir_v20i5e193_app3.pdf]

Multimedia Appendix 3. Correlation matrix of practice nurse (PN) characteristics

	1	2	3	4	5	6	7	8	9	10	11	12	13
1. Working hours	1.00	.06	.07	-.02	-.03	-.02	-.03	-.01	.10	-.06	-.02	-.00	.10
2. Counseling experience		1.00	-.02	.00	.08	-.01	-.10	.03	-.02	-.05	-.07	.02	*.16*
3. Designated consulting-hours			1.00	.01	-.03	.09	.10	-.03	.07	*.13*	.09	.07	.10
4. Intention to use any evidence-based guideline				1.00	*.41*	-.00	*.33*	*-.39*	*.12*	*.31*	*.29*	*.31*	*.12*
5. Intention to use STIMEDIC®					1.00	.07	*.23*	*-.17*	.11	*.18*	*.13*	*.16*	.04
6. STIMEDIC® knowledge						1.00	.10	-.08	.01	-.01	.05	.06	-.05
7. Perceived advantages							1.00	*.40*	.11	*.29*	*.34*	*.40*	-.00
8. Perceived disadvantages								1.00	*-.30*	*-.30*	*-.33*	*-.33*	*-.17*
9. Self-efficacy									1.00	*.16*	*.17*	*.17*	*.14*
10. Social modelling										1.00	*.71*	*.69*	*.16*
11. Social support											1.00	*.88*	*.20*
12. Social norms												1.00	*.21*
13. Baseline guideline adherence													1.00

Note: Data in *italics* denote a significant correlation.
